# Supplementary material for: Contributions of the Four Essential Entry Glycoproteins to HSV-1 Tropism and the Selection of Entry Routes
Source: mBio. 2021 Mar 2;12(2):e00143-21. doi: 10.1128/mBio.00143-21 (PMC8092210; doi:10.1128/mBio.00143-21)
Supplement: FIG S1 [file mBio.00143-21-sf001.pdf]

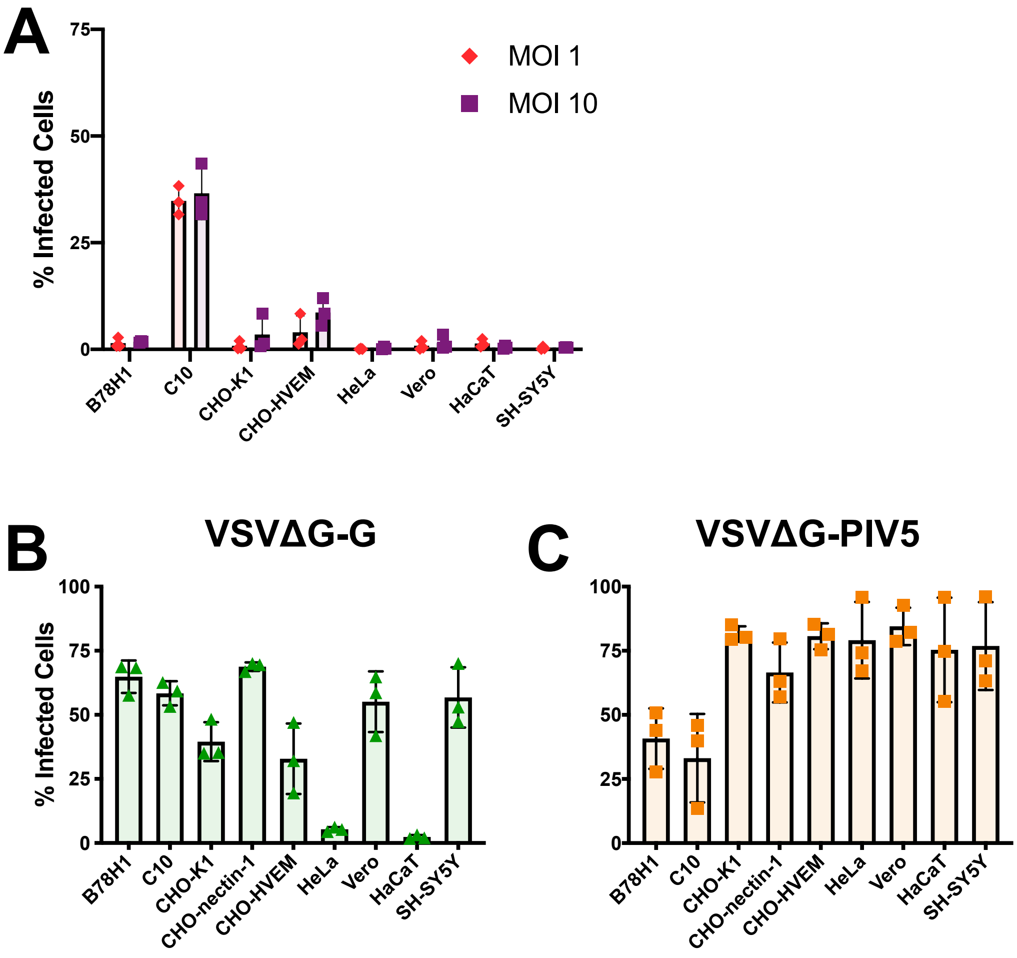


**Fig S1. Infecting cells with VSVΔG-BHLD at a higher MOI does not increase entry to an appreciable extent.** A)Receptor null (B78H1 and CHO-K1) and receptor bearing cells (C10, CHO-HVEM, HeLa, Vero, HaCaT, and SH-SY5Y) were infected at MOI =1 (red) or MOI = 10 (purple). Entry efficiency was assessed by flow cytometry at 6 hours post infection. B and C) Receptor null (B78H1 and CHO-K1) and receptor bearing cells (C10, CHO-HVEM, HeLa, Vero, HaCaT, and SH-SY5Y) were infected at MOI =1 with either VSVΔG-G (B) or VSVΔG-PIV5 (C). Entry was assessed by flow cytometry at 6 hours post infection.
