# Supplementary material for: Contributions of the Four Essential Entry Glycoproteins to HSV-1 Tropism and the Selection of Entry Routes
Source: mBio. 2021 Mar 2;12(2):e00143-21. doi: 10.1128/mBio.00143-21 (PMC8092210; doi:10.1128/mBio.00143-21)
Supplement: FIG S2 [file mBio.00143-21-sf002.pdf]

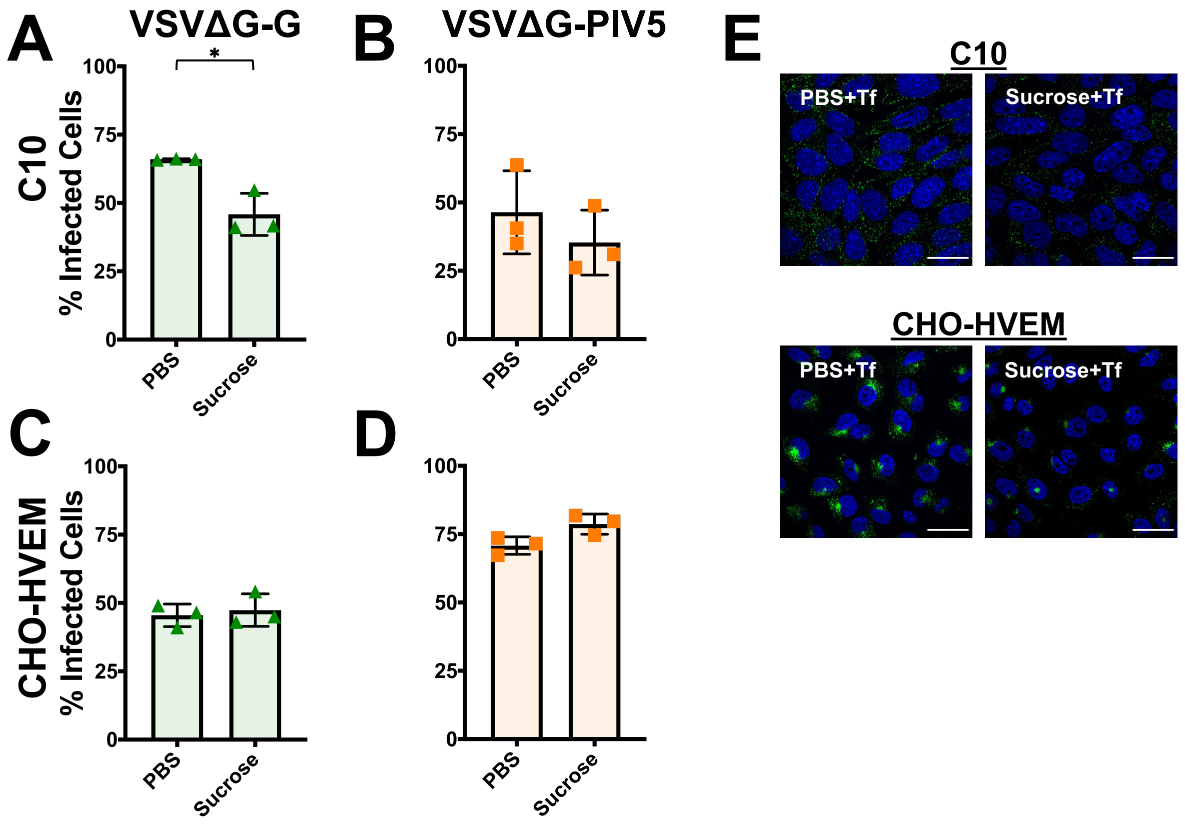

Fig. S2. VSVΔG-G and VSVΔG-PIV5 entry in the presence of hypertonic sucrose. C10 (A, B) and CHO-HVEM (C, D) cells were pretreated with a hypertonic solution of sucrose (0.3 M) and infected with VSVΔG-G or VSVΔG-PIV5 at MOI = 1. Infectivity was quantitated by flow cytometry at 6 hours post infection. Significance was calculated using a two-tailed Student's T-test with Welch's correction ($p < 0.05$ = *; $p < 0.01$ = **; $p < 0.001$ = ***). E) C10 and CHO-HVEM cells were pretreated with 0.3 M sucrose and incubated with 50 ug/ml of AF488-labeled transferrin (Tf). Cells were fixed, counterstained with DAPI, and imaged by confocal microscopy. Scale bar = 25 μm.
