# Supplementary material for: Contributions of the Four Essential Entry Glycoproteins to HSV-1 Tropism and the Selection of Entry Routes
Source: mBio. 2021 Mar 2;12(2):e00143-21. doi: 10.1128/mBio.00143-21 (PMC8092210; doi:10.1128/mBio.00143-21)
Supplement: FIG S3 [file mBio.00143-21-sf003.pdf]

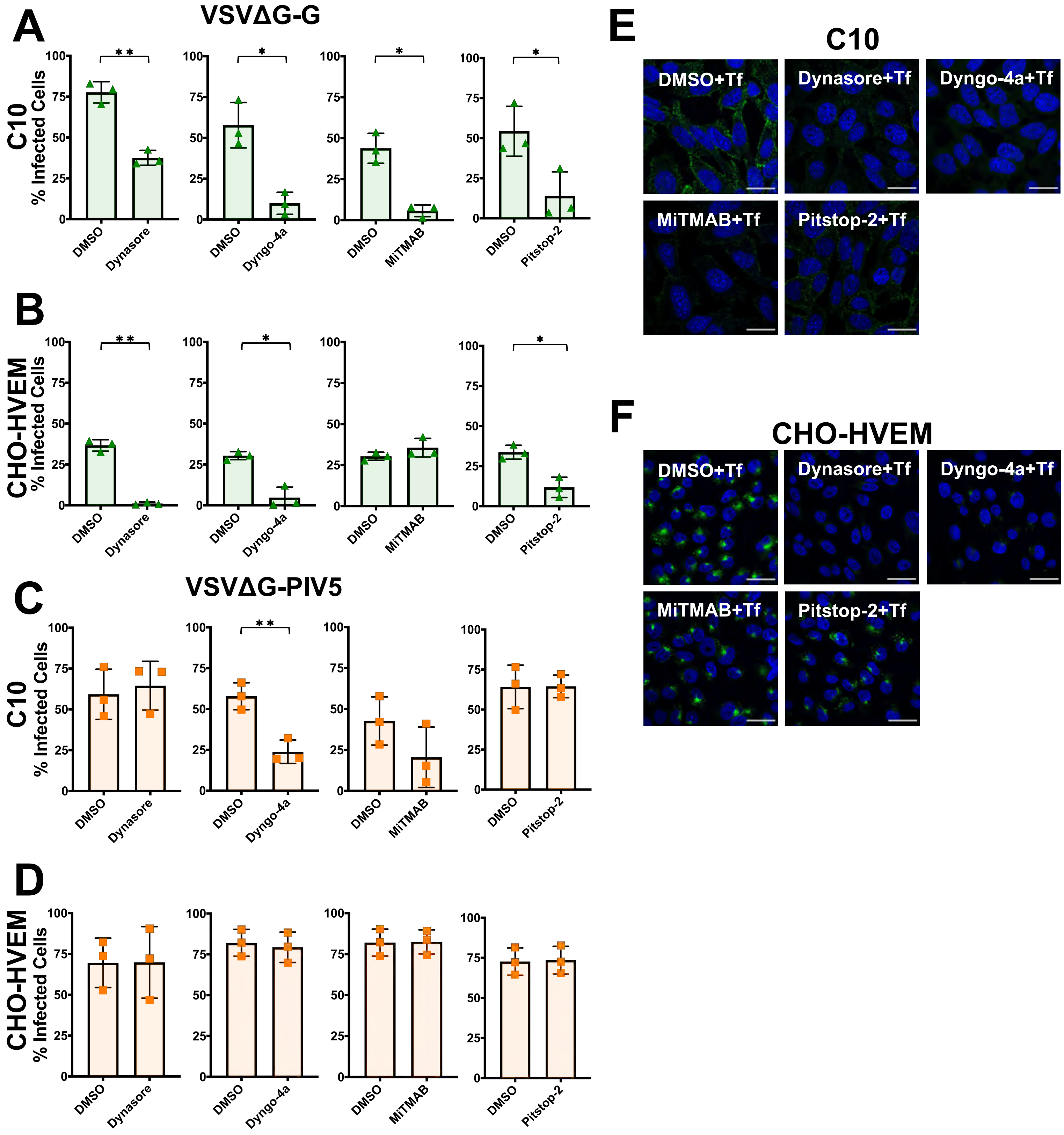

Fig. S3 VSVΔG-G and VSVΔG-PIV5 differ in their dependence on dynamin and clathrin for entry. C10 (A and C) and CHO-HVEM (B and D) cells were pretreated with dynamin inhibitors Dynasore (80 μM), Dyngo-4a (25 μM), MiTMAB (5 μM), or the CME inhibitor Pitstop-2 (30 μM) and infected with VSVΔG-G or VSVΔG-PIV5 at a MOI of 1. Infectivity was quantitated by flow cytometry at 6 hours post infection. CHO-HVEM cells treated with Dyngo-4a or MiTMAB used the same DMSO control as indicated by the same bar graph appearing twice each in panels C and D. Significance was calculated using a two-tailed Student's T-test with Welch's correction ($p < 0.05$ = *; $p < 0.01$ = **; $p < 0.001$ = ***). E and F) C10 and CHO-HVEM cells were pretreated with dynamin inhibitors Dynasore, Dyngo-4a, MiTMAB, or Pitstop-2 at the same concentrations as in panels A-D and then incubated with 50 μg/ml of AF488-labeled transferrin. Cells were fixed, counterstained with DAPI, and imaged by confocal microscopy. Scale bar = 25 μm.
