# Supplementary material for: Contributions of the Four Essential Entry Glycoproteins to HSV-1 Tropism and the Selection of Entry Routes
Source: mBio. 2021 Mar 2;12(2):e00143-21. doi: 10.1128/mBio.00143-21 (PMC8092210; doi:10.1128/mBio.00143-21)
Supplement: FIG S4 [file mBio.00143-21-sf004.pdf]

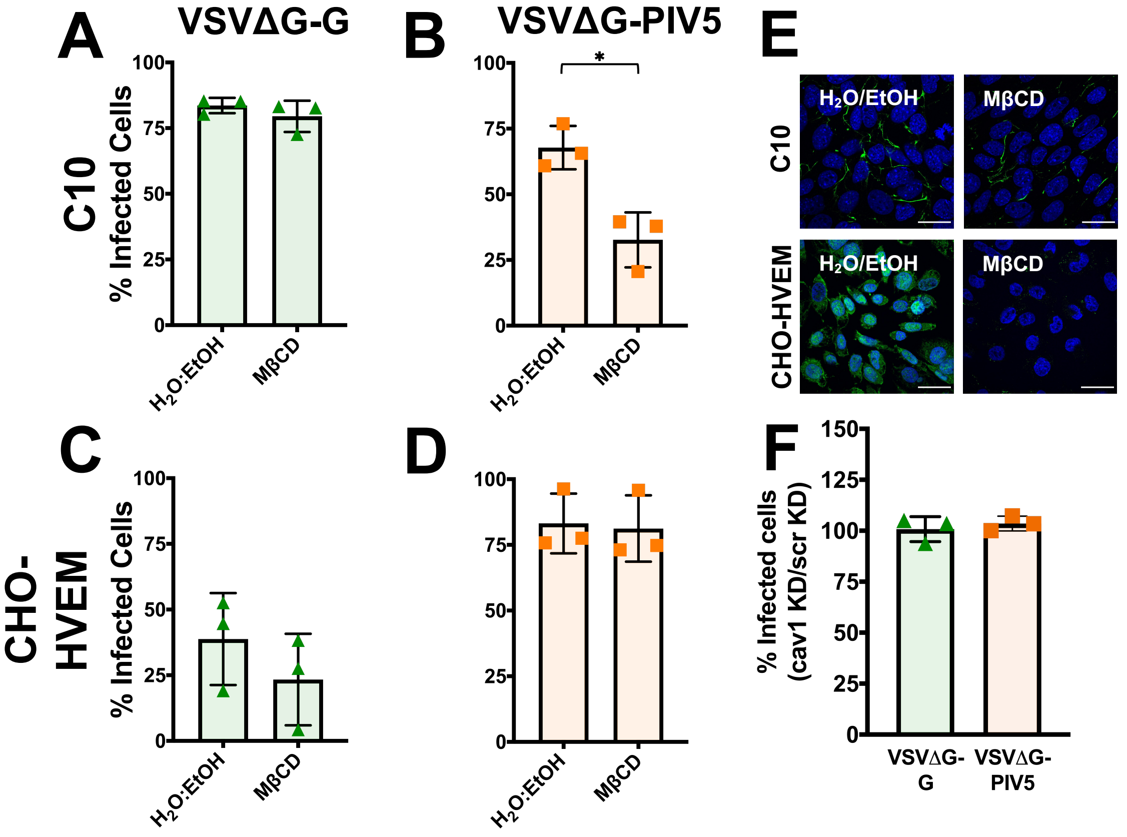

Fig. S4. VSVΔG-G entry does not require cholesterol whereas VSVΔG-PIV5 entry requires cholesterol in a cell-type-dependent manner. C10 (A and B) and CHO-HVEM (C and D) cells were pretreated with a cholesterol-removal drug methyl-β-cyclodextran, MβCD (5 mM) and infected with VSVΔG-G or VSVΔG-PIV5 at a MOI of 1. Infectivity was quantitated by flow cytometry at 6 hours post infection. E) C10 and CHO-HVEM cells were treated with either a solvent control (H_2O/EtOH) or methyl-β-cyclodextrin (MβCD), then incubated with cholera toxin subunit B labelled with Alexa Fluor 488. Confocal microscopy was performed on the solvent control and methyl-β-cyclodextrin treated cells. Cells were fixed, counterstained with DAPI, and imaged by confocal microscopy. Scale bar = 25 μm. (F) CHO-HVEM cells were transfected with a caveolin-1 siRNA (cav-1) or a scrambled control siRNA (scr) (both 50 pm) and infected with VSVΔG-G or VSVΔG-PIV5 at a MOI of 1. Infectivity was quantitated by flow cytometry at 6 hours post infection. Significance was calculated using a two-tailed Student's T-test with Welch's correction ($p < 0.05$ = *; $p < 0.01$ = **; $p < 0.001$ = ***).
