# Supplementary material for: Contributions of the Four Essential Entry Glycoproteins to HSV-1 Tropism and the Selection of Entry Routes
Source: mBio. 2021 Mar 2;12(2):e00143-21. doi: 10.1128/mBio.00143-21 (PMC8092210; doi:10.1128/mBio.00143-21)
Supplement: FIG S6 [file mBio.00143-21-sf006.pdf]

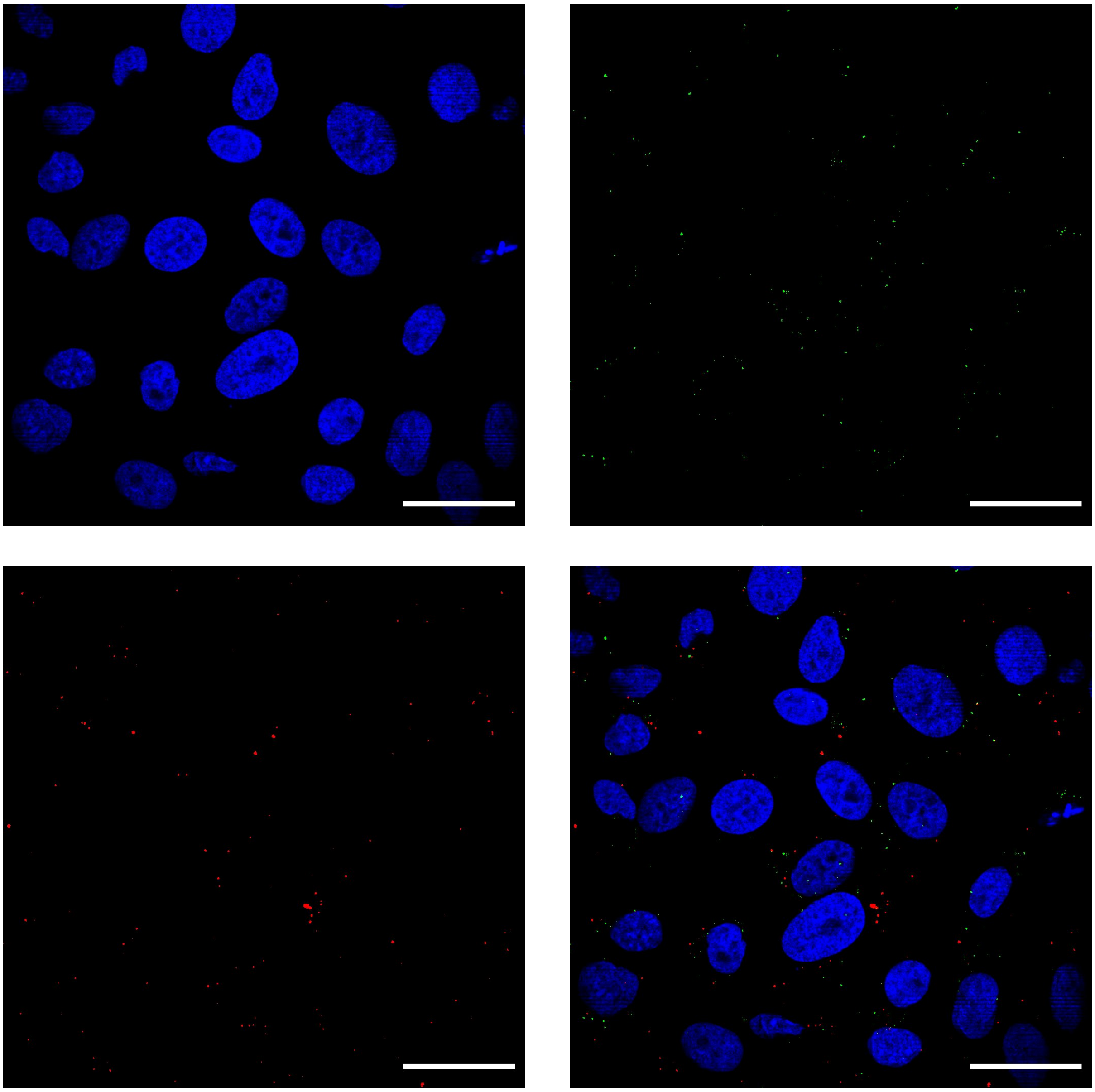

Fig. S6. VSVΔG-BHLD, in large, does not co-localized with the fluid-phase marker, 70 kDa dextran, in CHO-HVEM cells. CHO-HVEM cells were incubated with 1 mg/ml of rhodamine-B labelled 70 kDa dextran and VSVΔG-BHLD (MOI = 1) for one hour at 4˚C. Cells were then shifted to 37˚C for 20 minutes. Cells were fixed, counterstained with DAPI, and imaged by confocal microscopy. gB was detected by immunofluorescence using the rabbit pAb R68 and anti-rabbit IgG conjugated to FITC. Green = gB (marker for VSVΔG-BHLD particles); Red = 70 kDa dextran. Scale bar = 25 μm.
