# Supplementary material for: Contributions of the Four Essential Entry Glycoproteins to HSV-1 Tropism and the Selection of Entry Routes
Source: mBio. 2021 Mar 2;12(2):e00143-21. doi: 10.1128/mBio.00143-21 (PMC8092210; doi:10.1128/mBio.00143-21)
Supplement: FIG S7 [file mBio.00143-21-sf007.pdf]

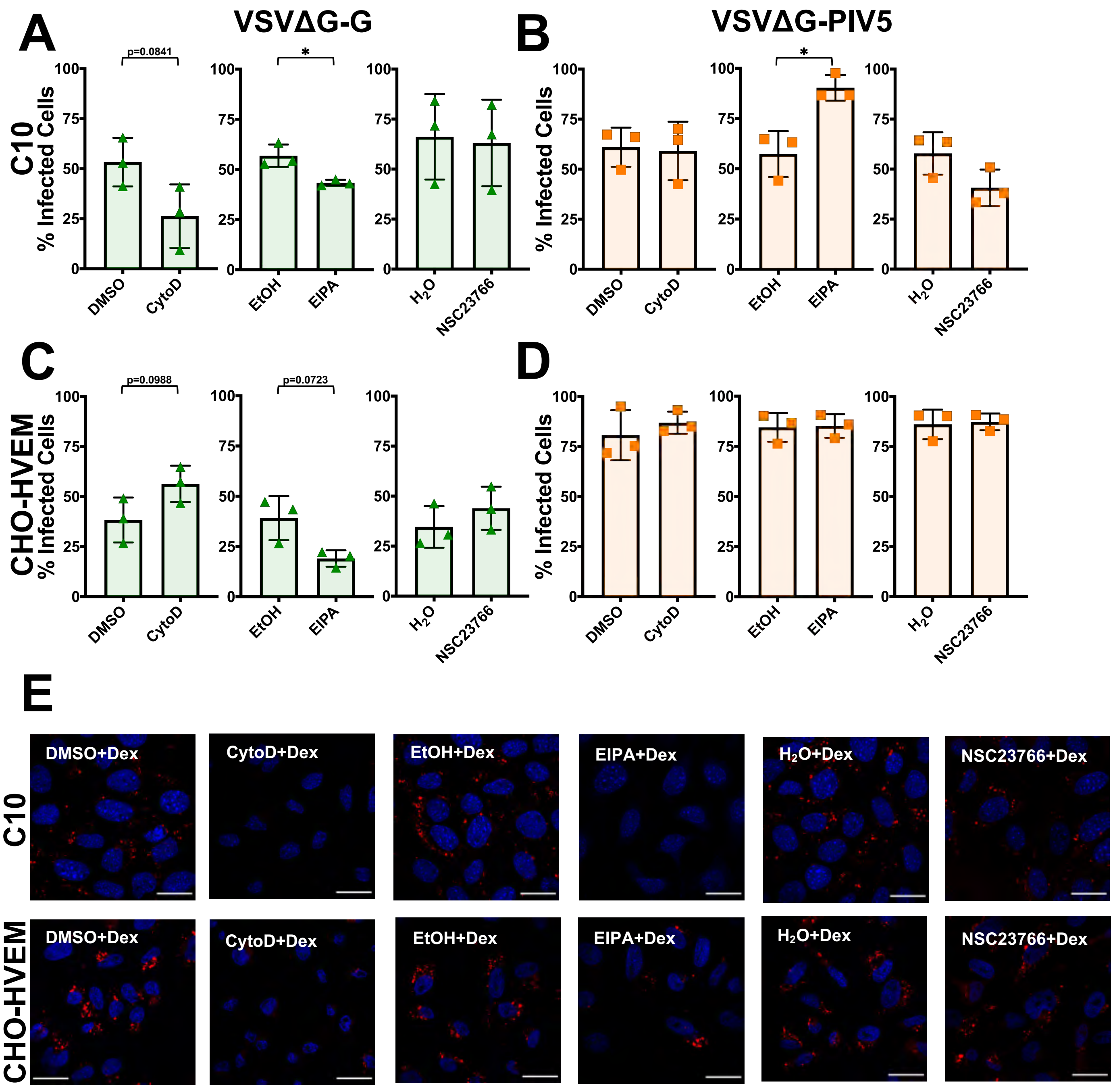

Fig. S7. VSVΔG-G and VSVΔG-PIV5 entry does not require macropinocytosis. C10 (A and B) and CHO-HVEM (C and D) cells were pretreated with macropinocytosis inhibitors cytochalasin D (2 μM), EIPA (25 μM), or NSC23766 (200 μM) and infected with VSVΔG-G or VSVΔG-PIV5 at a MOI of 1. Infectivity was quantitated by flow cytometry at 6 hours post infection. Significance was calculated using a two-tailed Student's T-test with Welch's correction ($p < 0.05$ = *; $p < 0.01$ = **; $p < 0.001$ = ***). E) C10 and CHO-HVEM cells were pretreated with macropinocytosis inhibitors cytochalasin D, EIPA, or NSC23766 at the same concentrations as in panels A-D and then incubated with 1.0 mg/ml of Rhodamine-B-labeled 70-kDa dextran (Dex). Cells were fixed, counterstained with DAPI, and imaged by confocal microscopy. Scale bar = 25 μm.
