# Supplementary material for: Contributions of the Four Essential Entry Glycoproteins to HSV-1 Tropism and the Selection of Entry Routes
Source: mBio. 2021 Mar 2;12(2):e00143-21. doi: 10.1128/mBio.00143-21 (PMC8092210; doi:10.1128/mBio.00143-21)
Supplement: FIG S8 [file mBio.00143-21-sf008.pdf]

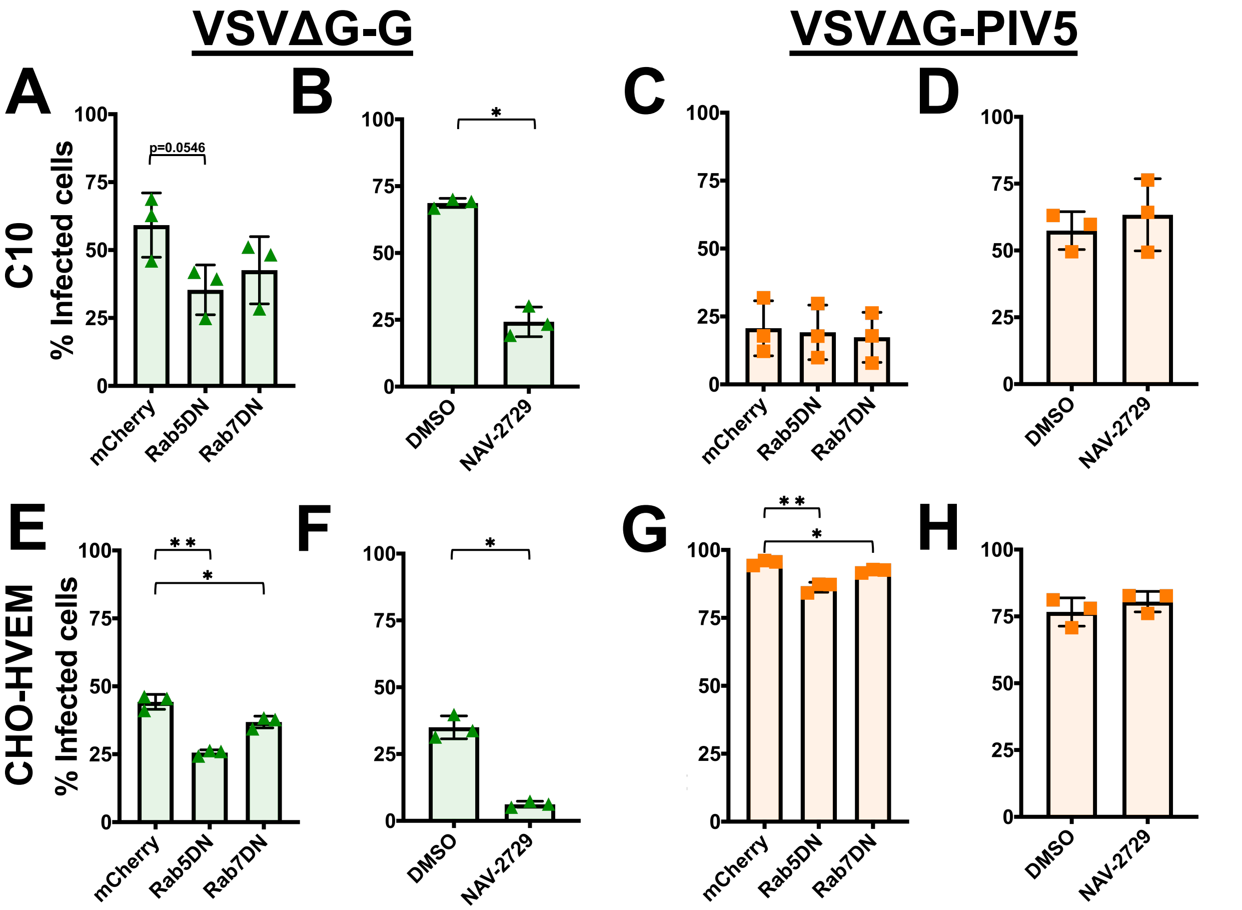

Fig. S8: **Roles of Rab5, Rab7, and Arf6 in VSVΔG-G and VSVΔG-PIV5 entry.** The roles of the small GTPases Rab5, Rab7 (A, C, E, G), and Arf6 (B, D, F, H) were assessed for VSVΔG-G (A, B, E, F) and VSVΔG-PIV5 (C, D, G, H) entry into C10 (A, B, C, D) and CHO-HVEM (E, F, G, H) cells. C10 (A and C) and CHO-HVEM (E and G) cells were transfected with either an empty vector control (eGFP or mCherry), eGFP or mCherry-tagged Rab5 dominant negative (DN), or eGFP or mCherry-tagged Rab7DN. Cells were infected at an MOI = 1 with either VSVΔG-G or VSVΔG-PIV5. Entry was assessed by flow cytometry at 6 hpi. The percent of infected cells was determined by dividing the number of virus(+)/eGFP/mCherry(+) cells by the total number of eGFP/mCherry(+) cells. C10 (B and D) and CHO-HVEM cells (F and H) were treated with the Arf6 inhibitor NAV-2729 (25 μM) and infected with either VSVΔG-G or VSVΔG-PIV5 at an MOI = 1. Significance was calculated using a two-tailed Student's T-test with Welch's correction (ns = not significant; $p < 0.05$ = *; $p < 0.01$ = **; $p < 0.001$ = ***).
