# Supplementary material for: Contributions of the Four Essential Entry Glycoproteins to HSV-1 Tropism and the Selection of Entry Routes
Source: mBio. 2021 Mar 2;12(2):e00143-21. doi: 10.1128/mBio.00143-21 (PMC8092210; doi:10.1128/mBio.00143-21)
Supplement: FIG S9 [file mBio.00143-21-sf009.pdf]

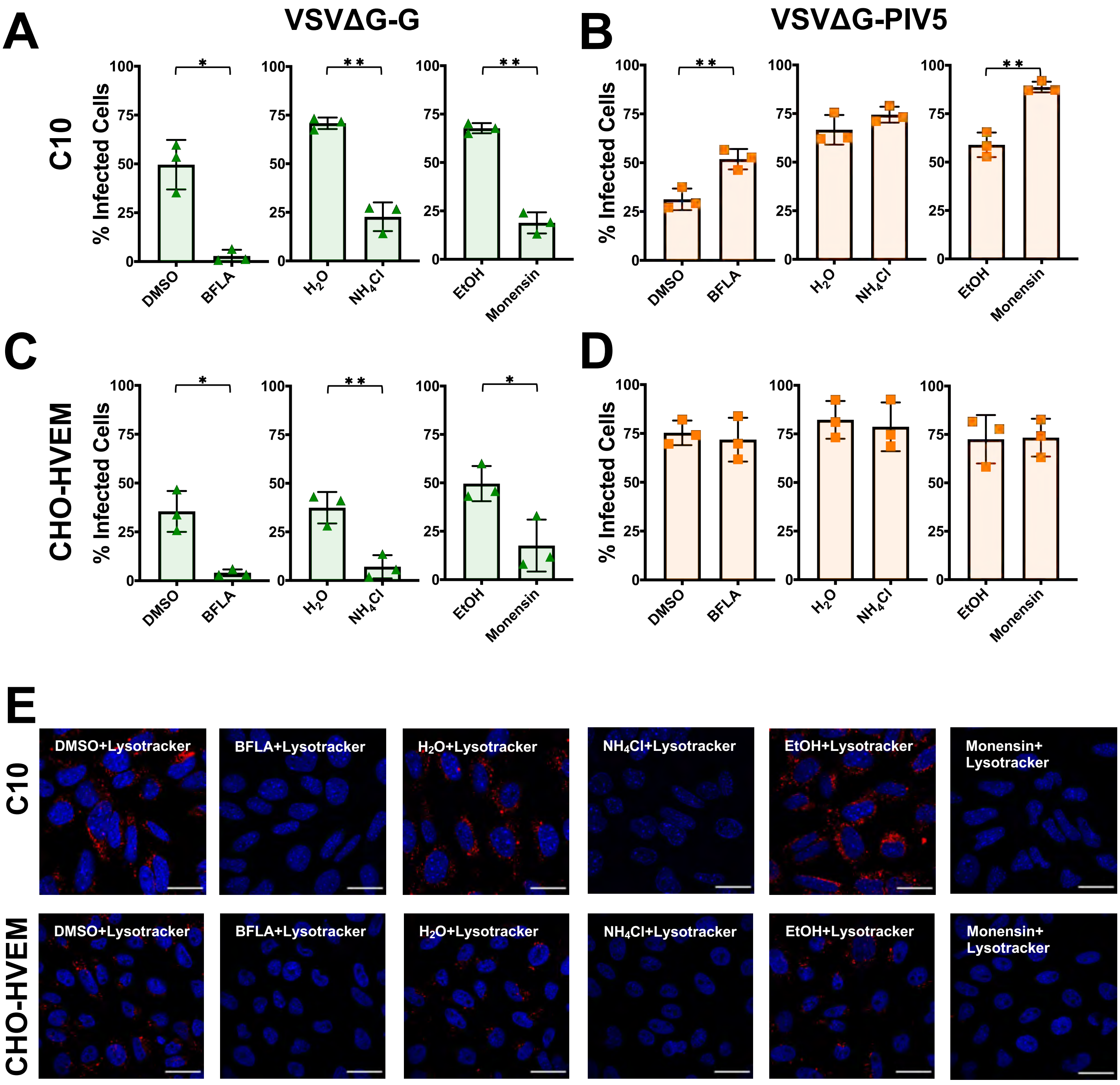

Fig. S9. VSVΔG-G but not VSVΔG-PIV5 entry requires endosomal acidification. C10 (A and B) and CHO-HVEM (C and D) cells were pretreated with inhibitors of endosomal acidification BFLA (100 nM), NH_4Cl (50 mM), or monensin (15 μM) and infected with VSVΔG-G or VSVΔG-PIV5 at MOI = 1. Infectivity was quantitated by flow cytometry at 6 hours post infection. Significance was calculated using a two-tailed Student's T-test with Welch's correction ($p < 0.05$ = *; $p < 0.01$ = **; $p < 0.001$ = ***). E) C10 and CHO-HVEM cells were pretreated with inhibitors of endosomal acidification at the same concentrations as in panels A-D (BFLA, NH_4Cl, or monensin) and then incubated with Lysotracker (1 μM). Cells were fixed, counterstained with DAPI, and imaged by confocal microscopy. Scale bar = 25 μm.
