# Supplementary material for: Contributions of the Four Essential Entry Glycoproteins to HSV-1 Tropism and the Selection of Entry Routes
Source: mBio. 2021 Mar 2;12(2):e00143-21. doi: 10.1128/mBio.00143-21 (PMC8092210; doi:10.1128/mBio.00143-21)
Supplement: TABLE S1 [file mBio.00143-21-st001.pdf]

Target	Inhibitors	HSV-1		VSVΔG-BHLD		VSVΔG-G		VSVΔG-PIV5	
		C10	CHO-HVEM	C10	CHO-HVEM	C10	CHO-HVEM	C10	CHO-HVEM
Endocytosis	Hypertonic sucrose	✓	✓	✓	✓	✓	X	X	X
Dynamin	Dynasore	✓	✓	✓	✓	✓	✓	X	X
	Dyngo-4a	✓	✓	✓	✓	✓	✓	✓	X
	MiTMAB	✓	✓	✓	✓	✓	✓	X	X
Clathrin	Pitstop-2	✓	✓	X	X	✓	✓	X	X
Cholesterol	MβCD	✓	✓	✓	✓	X	X	✓	X
Caveolin	Cav-1 siRNA	X	X	X	X	X	X	X	X
Macropinocytosis	CytoD	X	X	X	X	✓	X	X	X
	EIPA	X	✓	X	X	✓	✓	X	X
	NSC23766	X	X	✓	✓	X	X	X	X
Small GTPases	Rab5DN	X	X	✓	X	✓	✓	X	X
	Rab7DN	X	X	X	X	X	X	X	X
	NAV-2729	✓	✓	X	X	✓	✓	X	X
Low pH	BFLA	X	X	✓	X	✓	✓	X	X
	NH_4Cl	✓	✓	✓	✓	✓	✓	X	X
	Monensin	✓	✓	✓	X	✓	✓	X	X

Table S1. Sensitivity of HSV-1, VSVΔG-BHLD, VSVΔG-G, and VSVΔG-PIV5 to specific inhibitors. Green check marks indicate that virus entry is sensitive to that particular inhibitor. Red X marks indicate that the virus is not sensitive to that particular inhibitor.
